# Biophysical Characterization of CD6—TCR/CD3 Interplay in T Cells

**DOI:** 10.3389/fimmu.2018.02333

**Published:** 2018-10-09

**Authors:** Marjolein B. M. Meddens, Svenja F. B. Mennens, F. Burcu Celikkol, Joost te Riet, Johannes S. Kanger, Ben Joosten, J. Joris Witsenburg, Roland Brock, Carl G. Figdor, Alessandra Cambi

**Affiliations:** ^1^Department of Tumor Immunology, Radboud Institute for Molecular Life Sciences, Radboud University Medical Center, Nijmegen, Netherlands; ^2^Department of Cell Biology, Radboud Institute for Molecular Life Sciences, Radboud University Medical Center, Nijmegen, Netherlands; ^3^Department of Nano-BioPhysics, MIRA Institute for Biomedical Technology and Technical Medicine, University of Twente, Enschede, Netherlands; ^4^Department of Biochemistry, Radboud Institute for Molecular Life Sciences, Radboud University Medical Center, Nijmegen, Netherlands

**Keywords:** T cell, immunological synapse, T-cell receptor (TCR), CD3, CD6, membrane receptor, receptor dynamics

## Abstract

Activation of the T cell receptor (TCR) on the T cell through ligation with antigen-MHC complex of an antigen-presenting cell (APC) is an essential process in the activation of T cells and induction of the subsequent adaptive immune response. Upon activation, the TCR, together with its associated co-receptor CD3 complex, assembles in signaling microclusters that are transported to the center of the organizational structure at the T cell-APC interface termed the immunological synapse (IS). During IS formation, local cell surface receptors and associated intracellular molecules are reorganized, ultimately creating the typical bull's eye-shaped pattern of the IS. CD6 is a surface glycoprotein receptor, which has been previously shown to associate with CD3 and co-localize to the center of the IS in static conditions or stable T cell-APC contacts. In this study, we report the use of different experimental set-ups analyzed with microscopy techniques to study the dynamics and stability of CD6-TCR/CD3 interaction dynamics and stability during IS formation in more detail. We exploited antibody spots, created with microcontact printing, and antibody-coated beads, and could demonstrate that CD6 and the TCR/CD3 complex co-localize and are recruited into a stimulatory cluster on the cell surface of T cells. Furthermore, we demonstrate, for the first time, that CD6 forms microclusters co-localizing with TCR/CD3 microclusters during IS formation on supported lipid bilayers. These co-localizing CD6 and TCR/CD3 microclusters are both radially transported toward the center of the IS formed in T cells, in an actin polymerization-dependent manner. Overall, our findings further substantiate the role of CD6 during IS formation and provide novel insight into the dynamic properties of this CD6-TCR/CD3 complex interplay. From a methodological point of view, the biophysical approaches used to characterize these receptors are complementary and amenable for investigation of the dynamic interactions of other membrane receptors.

## Introduction

T cells play an important role in the execution of the adaptive immune response by regulating the activity of innate and other adaptive immune cells or directly executing effector functions, such as killing by cytotoxic T cells. In general, for T cells to execute their function, they need to become activated by antigens through interaction with an antigen-presenting cell (APC). Crucial to this activation is the interaction between the T cell receptor (TCR) on the T cell surface and the peptide-Major Histocompatibility Complex (pMHC) on the APC surface. Immediately after recognition of the pMHC, the TCR, associated with the CD3 receptor complex, combines with co-stimulatory receptors CD4/CD8 and CD28 on the T cell membrane in small so-called TCR microclusters where signaling is initiated (1, 2). During the T cell-APC contact, TCR microclusters are laterally transported during local cell surface receptor rearrangement creating a typical bull's eye-shaped pattern at the T cell-APC interface, termed “the immunological synapse” (IS) (3, 4). This lateral TCR microcluster transport results in TCR accumulation in the center of the IS, forming the central supramolecular activation cluster or “cSMAC,” together with co-stimulatory molecules such as CD2, CD4/CD8 and CD28 (4–6). Surrounding the central cSMAC is the peripheral supramolecular activation cluster (pSMAC), that exists of adhesion receptor LFA-1 and phosphatase CD45, both kept from the cSMAC due to size-dependent exclusion (7). This spatial organization of the receptors, together with the transport of TCR microclusters toward the cSMAC is dependent on the actomyosin cytoskeleton, which is excluded from the cSMAC region (8–11). Antigen binding on the extracellular side leads intracellularly to recruitment of tyrosine kinase Lck to the TCR/CD3 complex, where it phosphorylates immunoreceptor tyrosine-based activation motifs (ITAMs) on the cytoplasmic tail of CD3 chains (12). Subsequently, tyrosine kinase ZAP70 can bind to the phosphorylated ITAM-motifs and recruit the transmembrane protein LAT (12, 13). LAT forms a signaling hub, the so-called LAT signalosome, which through various signaling molecules such as SLP-76 and GRB2, initiates downstream events, such as calcium fluxing, actin reorganization, integrin inside-out signaling and gene expression, leading to T cell activation and effector functions (12, 14).

CD6 is one of the cell surface co-receptors on the T cell membrane involved in T cell activation. CD6 is a transmembrane glycoprotein, part of the scavenger receptor cysteine-rich superfamily (SRCR-SF), that is expressed on thymocytes, mature T cells, a subset of B cells and NK cells, and brain parenchymal cells (15–18). On the T cell membrane, CD6 associates with its closely related family member CD5 (17, 19). Extracellularly, ligands for CD6 are Activated Leukocyte Cell Adhesion Molecule (ALCAM), which is present on antigen presenting cells and thymic epithelial cells, and the recently identified CD318, a glycoprotein expressed on epithelial cells, some hematopoietic cells and mesenchymal stem cells (16, 20–22).

Already early on, it was clear that CD6 was involved in T cell activation in mature T cells, since monoclonal antibodies targeting CD6 were able to induce T cell activation, subsequent T cell proliferation and IL-2 receptor expression (23, 24). Since then, multiple studies have further substantiated a co-stimulatory role of CD6 in T cell activation (25–29). However, more recently this view was challenged by data from Oliveira and colleagues, who describe a role for CD6 as attenuator of early and late T cell responses in a ligand-independent manner (30). The exact role for CD6 in T cell signaling is therefore still under debate and most likely depends on a balance between stimulatory and inhibitory signals, provided among others by binding of its ligand (30, 31).

Multiple data hint at an interaction, either direct or indirect, between CD6 and the TCR. Co-precipitation studies have indicated that rat CD6 associates with protein kinases Lck, Fyn, ZAP-70, and Itk: protein kinases that also interact with and co-precipitate with the TCR or are part of the LAT signalosome (14, 32). This interaction is important for CD6 signaling, as inhibition of protein kinases abolishes CD6-induced T cell proliferation (26). Furthermore, CD6 physically associates with adaptor protein SLP-76 (33), which is involved in TCR microcluster signaling. Also, direct cross-linking of CD3 induces phosphorylation of CD6, which suggests cross-talk between TCR/CD3 complex and CD6 (34). More importantly, using co-precipitation Gimferrer and colleagues showed that CD6 and the TCR/CD3 complex interact (independently of CD5) (35). Also, co-localization of CD6 and TCR/CD3 in the cSMAC of the mature IS has been described through co-capping, FRET and DC-T cell co-cultures (29, 35). CD6 is important for mature IS formation as treatment with soluble recombinant CD6 leads to inhibition of IS maturation and resulted in inhibition of T cell proliferation (35).

Importantly, CD6 has recently reclaimed attention as a focus of research: the *CD6* gene, together with the gene for its ligand ALCAM, was identified as a susceptibility locus and a potential target for treatment of multiple sclerosis (36, 37). Furthermore, antibodies targeting CD6 are tested for treatment of various autoimmune diseases, such as psoriasis and rheumatoid arthritis (38–41). This renewed interest in CD6 underlines the importance of understanding CD6 signaling and interaction at the molecular level. For instance, although static co-localization of CD6 and TCR/CD3 complexes has been reported at the fully mature IS and signaling cross-talk between CD6 and CD3 has been identified, thorough characterization of (early) dynamics during IS formation and stability of CD6-TCR/CD3 interplay at the mature IS are still lacking.

Imaging techniques with high spatiotemporal resolution, such as Total Internal Reflection Fluorescence (TIRF) Microscopy, combined with biochemical or immunological assays, such as supported lipid bilayers (42), have been fundamental in unraveling the dynamics of multiple protein-protein interactions during IS formation (1, 11, 13). Here, we exploited different biophysical approaches including microcontact printing, fluorescence microscopy techniques, antibody-coated beads and magnetic tweezers to study the dynamics and stability of CD6-TCR/CD3 interplay in more detail. Overall, our findings provide novel insight into the dynamic properties of CD6—TCR/CD3 complex interplay during IS formation.

## Materials and methods

### Cell lines and transfection

Jurkat E6.1 lymphoma T cells were maintained in 1640 RPMI (PAA) supplemented with 10% Fetal Calf Serum (Greiner Bio-one), 1 mM Ultra-glutamine (U-glut, PAA) and antibiotics (100 U/ml penicillin, 100 μg/ml streptomycin and 0.25 μg/ml amphotericin B, PAA). Jurkat cell lines stably expressing CD6-RFP, CD6-GFP, or LifeAct-GFP were obtained by electroporation using the Neon Transfection System for Electroporation (Invitrogen) according to the manufacturer's guidelines. Shortly, 5^*^10^5^ Jurkat cells were transfected at 1325 Volt (10 ms, 3 pulses) with 3 μg of DNA in 100 μl Resuspension buffer. After transfection cells were seeded in 2 ml of 1640 RPMI with 10% FCS and 1% U-glut. Antibiotics were added after 3 h. Stable cell lines were sorted on RFP or GFP expression on a FACSAria cell sorter (BD Biosciences), and cells were maintained in complete RPMI medium as described above, additionally supplemented with 500 ng/ml geneticin (G418, Gibco).

### Antibodies, reagents and expression constructs

The following primary antibodies were used: Mouse IgG2A-anti-human CD3 antibodies clone T3B and clone OKT-3 (both referred to in the text as αCD3), and Mouse IgG1 anti-human LFA-1 antibody TS2/4 were obtained from in-house hybridoma production. Mouse IgG1 anti-human phospho-tyrosine (P-Tyr-100), both unconjugated and conjugated to Alexa488, was obtained from Cell Signaling Technology; Mouse IgG1 anti-human CD6 (M-T605; referred to in the text as αCD6) was obtained from BD Biosciences. The following secondary antibodies were used: Goat anti-Rabbit-IgG(H+L)-Alexa647 and Goat-anti-Mouse-IgG1-Alexa488 (both from Invitrogen). Neutravidin-TexasRed was obtained from Thermo Fisher Scientific. For use in immunofluorescence staining, anti-CD3 antibody clone OKT-3 was biotinylated (Sulfo-NHS-LC-Biotin, Thermo Fisher Scientific) at RT for 1.5 h, with a molecular ratio of IgG:Biotin at 1:15. Following the same procedure, for use in supported lipid bilayers, anti-human CD3 antibody OKT-3 was simultaneously biotinylated and conjugated to ATTO647 Carboxylic Acid, Succinimidyl ester (ATTO-TEC) at a molecular ratio of IgG:Biotin:dye at 1:15:15. In both cases, purification was performed with Zeba Desalting columns (Thermo Fisher Scientific). For preparation of supported lipid bilayers, the lipids POPC (1-palmitoyl-2-oleoyl-sn-glycero-3-phosphocholine) and Biotin Capped PE (1,2-Dioleoyl-sn-Glycero-3-Phosphoethanolamine-N-[Cap Biotinyl]), both from Avanti Polar Lipids Inc, were used, together with the fluorescent lipophilic tracer DiI (1,1′-Dioctadecyl-3,3,3′,3′-Tetramethylindocarbocyanine Perchlorate; Invitrogen). For inhibition of actin polymerization Cytochalasin D (CytoD) was used (2.5 μg/ml, Sigma-Aldrich). The CD6-GFP plasmid was generated by cloning CD6 from the CD6-RFP construct into peGFP-N1 (Clontech) (29). LifeAct-GFP (43) was a kind gift of Michael Sixt (Institute of Science and Technology, Vienna, Austria).

### Micro contact printing of antibody spots

PDMS stamps containing a regular pattern of 5 μm circular spots were prepared as described earlier (44). PDMS stamps were incubated for 1 h at RT with a protein solution containing 15 μg/ml Goat-anti-Rabbit IgG(H+L)-Alexa647 antibody to visualize the spots, and anti-human CD6 or anti-human CD3 clone T3B, the latter including (if indicated) mouse IgG2A isotype control antibody, to create spots containing 1 or 10% αCD3. Total concentration of primary antibody in the protein solution amounted to 100 μg/ml. After incubation, the stamps were thoroughly washed with distilled H_2_O and dried under a N_2_ stream. A glass microscope slide was cleaned by rinsing consecutively with distilled H_2_O, 70% ethanol and 100% acetone, and was dried under a N_2_ stream. The stamp was then manually pushed on the cleaned glass microscope slide for 20 s and removed, after which the stamped area was back-filled with 20 μg/ml fibronectin (from human plasma; Roche) in PBS for 1 h at RT. The microscope slide was washed in PBS and incubated with 1% BSA for 30 min to block all uncoated glass surface. The slide was subsequently washed with PBS and dried under a N_2_ stream before cell seeding.

### Preparation of supported lipid bilayers (SLBs)

Coverslips were cleaned in 2% v/v Hellmanex III (Hellma-Analytics) solution and sonified for 15 min at RT after which they were rinsed with ultra clean water and ethanol and dried under a N_2_ stream. SLBs were prepared by spin coating (45). To form SLBs, a lipid chloroform mixture containing 1 mM POPC, 0.01 mM Biotin Capped PE, supplemented with DiI, was dropped on a spinning coverslip. SLBs were hydrated with Hank's Balanced Salt Solution (HBSS; Gibco) throughout the preparation. After deposition, nonspecific binding was blocked by incubation with 10 mg/ml BSA in HBSS. Subsequently, SLBs were incubated with 0.5 μg/ml streptavidin (Thermo Fisher scientific). Finally, SLBs were incubated with 0.5 μg/ml biotinylated anti-human CD3 (OKT3)-ATTO647 for 15 min at RT, after which they were used directly for cell seeding.

### Immunofluorescence

Immunofluorescent staining of CD3 was performed on wildtype Jurkat T cells on microprinted antibody spots. Immunofluorescent staining of phospho-tyrosine was performed on wildtype Jurkat T cells on microprinted antibody spots and on wildtype Jurkat T cells on SLBs. LFA-1 staining was performed on wildtype Jurkat T cells on SLBs. Cells were seeded on spots or SLBs for 15–30 min at 37°C. Samples were washed with PBS and subsequently fixed with 4% PFA in PBS for 15 min at RT. After fixation, samples were blocked for 1 h with blocking solution (PBS/3% BSA/10 mM glycine/1% human serum) at RT. For CD3 and phospho-tyrosine staining on antibody spots and for phospho-tyrosine staining on SLBs, permeabilization was performed simultaneously with blocking by adding 0.1% saponin to the blocking solution. 0.1% saponin was added to all subsequent antibody staining solutions. For LFA-1 staining, after blocking, cells on SLBs were incubated with primary Mouse IgG1 anti-human LFA-1 antibody TS2/4 and subsequently Goat-anti-Mouse-IgG1-Alexa488. For phospho-tyrosine staining, after blocking/permeabilization, cells on antibody spots were incubated with Mouse IgG1 p-Tyr-100-Alexa488; cells on SLBs were incubated with primary Mouse IgG1 p-Tyr-100 and subsequently Goat-anti-Mouse-IgG1-Alexa488. For CD3 staining, after blocking/permeabilization, cells on antibody spots were incubated with OKT3-biotin and subsequently NeutrAvidin-Texas-Red. After immunofluorescence staining, samples on microprinted antibody spots were washed with phosphate buffer and embedded in Mowiol (Sigma-Aldrich). Immunofluorescence samples on SLBs were not embedded but imaged in PBS directly after preparation. To study the effect of inhibition of actin polymerization on IS formation, CD6-GFP Jurkat cells were taken from culture and incubated in HBSS with or without 0.5 μM Cytochalasin D for 15 min at 37°C at a concentration of 800,000 cells per ml. Next, cell suspensions were added onto αCD3-containing SLBs, reaching a final cell concentration of 400,000 cells per ml. Samples were incubated for 30 min at 37°C. After incubation, samples were fixed with 4% PFA in PBS for 15 min at RT. Finally samples were washed once and imaged in PBS directly after preparation. CD6-GFP, αCD3-ATTO647, DiI, and brightfield signals were acquired. Samples of cells seeded on microprinted antibody spots were imaged on an Olympus FV1000 confocal laser scanning microscope with a 60 × 1.35 NA oil immersion objective. Samples of cytochalasin D treated cells on SLBs were imaged using a Leica DMI6000 widefield microscope equipped with a HC PL APO 63 × 1.40 NA oil immersion objective. Samples of LFA-1 and phospho-tyrosine staining in cells on SLBs were imaged with TIRF microscopy at an Olympus IX-71 wide field fluorescence microscope equipped with a 3-line TIRF system and a Hamamatsu ImagEM EM-CCD camera equipped with a PL APO 60 × /1.4 NA oil immersion TIRF objective.

### Live cell imaging on SLBs

Live cell imaging in cells on SLBs was performed at 37°C with TIRF microscopy at the Olympus TIRF microscope setup described above. Prior to live cell imaging, Jurkat cells (LifeAct-GFP or CD6-GFP) were washed with PBS and resuspended in HBSS. Cells were added to the SLBs at the microscope, in a final concentration of 400,000 cells per ml HBSS. Directly after adding the cells, both cells and αCD3-ATTO647coupled to the SLBs were imaged. Images were acquired at a frame rate of 300 ms/frame or 1 s/frame with an exposure time of 10–100 ms.

### Cell-bead contact experiments

Dynal CD3 beads coated with mouse monoclonal anti-CD3 antibody (Invitrogen) or fibronectin-coated beads, all with a diameter of 4.5 μm, were used for bead experiments. Jurkat CD6-GFP cells were seeded on fibronectin-coated coverslips in imaging medium (RPMI 1640, 25 mM HEPES, 0.5% BSA). Subsequently, beads were added to the cells in a concentration of 5 μM. Imaging of the CD6-GFP signal and the brightfield channel of cells with beads was performed on a Zeiss LSM510 meta confocal laser scanning microscope equipped with a PL APO 63 × /1.4 NA oil immersion objective. Cells were imaged at RT to slow down internalization of the beads.

### FRAP measurements

All FRAP measurements were performed on a Zeiss LSM510 meta confocal laser scanning microscope equipped with a PL APO 63 × /1.4 NA oil immersion objective. For FRAP on antibody spots, Jurkat CD6-GFP cells were resuspended in phenol red-free medium, seeded onto microprinted surfaces and imaged at 37°C. FRAP was performed using a 2.1 μm diameter circular region of interest (ROI). Photobleaching was performed at 100% laser power by scanning the bleached ROI for two iterations, yielding a total bleach time of 0.10 s and an average fluorescence loss of ~50%. Recoveries were collected with time intervals of 200 ms using 488 nm excitation. Fluorescence intensity data for the bleached ROI and a control ROI were calculated using LSM software (Zeiss). After background correction and normalization to t_0_ using a method that is known as double normalization (46), the single post-bleach curves were fitted using Origin (OriginLab) with the following model:

$$\begin{array}{r} I\left(t\right)=A*e^{-t/\tau } \end{array}$$

where *I(t)* is the intensity in the bleached ROI at time *t, A* is the mobile fraction, and τ is the characteristic recovery time. The halftime recovery t_0.5_ was calculated with:

$$\begin{array}{r} t_{0.5}=\;ln2*\;\tau \end{array}$$

For FRAP measurements on cells in contact with beads, Jurkat CD6-GFP cells were resuspended in phenol red-free medium, incubated with αCD3-coated beads and seeded on fibronectin-coated surfaces. FRAP was performed using a 2 × 1 μm rectangular ROI. Photobleaching was performed at 100% laser power by scanning the bleached ROI for 20 iterations, yielding a total bleach time of 1 s and average fluorescence loss of ~50%. Recoveries were collected with time intervals of 100 ms using 488 nm excitation. After background correction and single normalization, FRAP curves were fitted using the Ellenberg fitting (47) with the help of FRAPAnalyser software (48):

$$\begin{array}{c} I\left(t\right)=I_{final}{\left(1-(w^{2}{\left(w^{2}+4\pi Dt\right)}^{-1}\right)}^{1/2} \end{array}$$

Where *I(t)* is the fluorescence intensity as a function of time, I_final_ the final intensity reached after complete recovery, *w* the width of the rectangular ROI, and *D* is the one-dimensional diffusion constant. Recovery halftime *t*_0.5_ was calculated using the formula (49):

$$\begin{array}{r} D\;=\frac{0.88{\text{w}}^{2}}{4{\text{t}}_{0.5}} \end{array}$$

### Image analysis

Image analysis was performed using Fiji Image J (50). To quantify the immunofluorescence images of cells on microprinted spots, a custom image analysis algorithm was used. Shortly, spots were segmented based on an intensity threshold applied to the spots channel. Cells were segmented using an edge finding algorithm applied to the DIC image and a selection of objects based on size. Next, the segmentations of the spots and cells were combined resulting in masks for cellular parts covering the spots and cellular parts covering the surrounding area. These masks were subsequently used to measure the intensity of the fluorescent protein or immunostaining on the spots and the surrounding area. Enrichment of CD6 was quantified by measuring the ratio of the fluorescent intensity in parts of the cells covering the spots and the fluorescent intensity in parts of the cells covering the surrounding area coated with fibronectin. A value of 1 indicates no recruitment, while values higher or lower than 1 indicate recruitment or exclusion, respectively. In Jurkat CD6-GFP cells on SLBs, kymographs were created along the indicated lines using the *Dynamic Reslice* option under *Image* > *Stacks* in FIJI Image J for the indicated time periods. Co-localization of CD6-GFP and αCD3 during immunological synapse formation was determined using the JACoP plugin in FIJI Image J (51). A ROI in the central part of the cell-SLB interface was selected and the same size ROI was applied to each time point and to all cells analyzed. Co-localization was quantified by determining the Mander's Coefficient M1 by making use of appropriate thresholding which only included CD6- and αCD3-rich microclusters or the cSMAC. Also the Mander's Coefficient M2 (with same thresholding as for M1) and the Pearson Coefficient over time were determined. Relative CD6-GFP signal intensity in the same ROI over time was determined by calculating the integrated density of the total ROI and relating it to the integrated density at *t* = 2 min (start of immunological synapse formation). The fraction of cells forming an immunological synapse on SLBs upon Cytochalasin D treatment was determined by manual counting. Cells having formed an immunological synapse were defined as CD6-GFP positive cells, also visible in brightfield, on top of SLB (DiI-positive area), overlaying an αCD3 positive cluster. In bead experiments, CD6 enrichment was determined as the ratio between CD6-GFP fluorescence intensity of the membrane area of cell that was in contact with the bead and the fluorescence intensity in an equal sized ROI in the membrane of the cell at the opposite side of bead contact.

### Statistical analysis

Statistical analysis was carried out with GraphPad Prism version 5.03. Data are presented as mean ± standard deviation for bar plots and median ± interquartile range for box plots. To compare two groups, a paired/unpaired *t-*test was applied. To compare three or more groups, one-way ANOVA with *post-hoc* Tukey's Multiple Comparison test or Kruskal–Wallis test with *post-hoc* Dunn's Multiple Comparison test was applied. Differences were considered statistically significant at *p* < 0.05.

## Results

### CD6 and TCR/CD3 in Jurkat T cells co-localize upon ligation through micropatterned antibody spots

**Figure 1:**
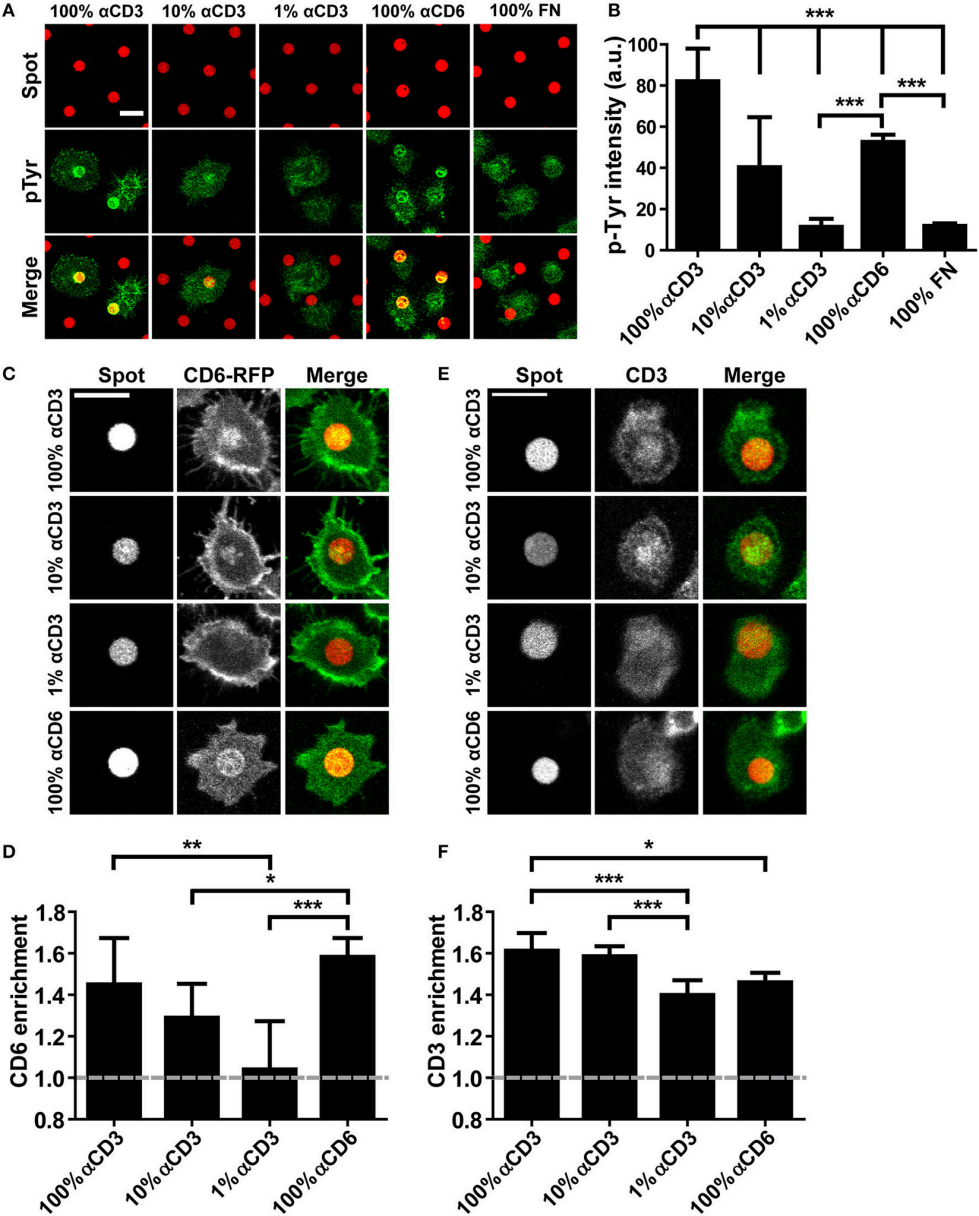
CD6 and TCR/CD3 in Jurkat T cells co-localize upon ligation through micropatterned antibody spots. **(A,B)** Wildtype Jurkat T cells were seeded on micropatterned substrates containing 100, 10, or 1% αCD3 spots, 100% αCD6 spots or 100% fibronectin (FN) spots, surrounded by fibronectin, fixed after 15 min and stained for phospho-tyrosine. All spots were labeled with Alexa647, phospho-tyrosine was labeled with Alexa488. Representative confocal images are shown in **(A)**, quantification of phospho-tyrosine intensity in the spot area of *n* = 10 cells per condition is shown in **(B)**. Bars represent mean with SD. Statistical significance was tested with one-way ANOVA with *post-hoc* Tukey's Multiple Comparison test. **(C,D)** CD6-RFP Jurkat T cells were seeded on micropatterned substrates containing 100, 10, or 1% αCD3 spots, or 100% αCD6 spots, surrounded by fibronectin, and fixed after 15 min. All spots were labeled with Alexa647. Representative confocal images are shown in **(C)**, quantification of CD6 enrichment of *n* = 10 cells per condition is shown in **(D)**. CD6 enrichment is defined as the ratio between CD6-RFP intensity of parts of the cell on the spot vs. part of the cell covering the surrounding fibronectin. Bars represent mean with SD. Statistical significance was tested with Kruskal–Wallis test with *post-hoc* Dunn's Multiple Comparison test. **(E,F)** Wildtype Jurkat cells were seeded on micropatterned substrates containing 100, 10, or 1% αCD3 spots, or 100% αCD6 spots, surrounded by fibronectin, fixed after 15 min and stained for CD3. All spots were labeled with Alexa647, CD3 was labeled with TexasRed. Representative confocal images are shown in **(E)**, quantification of CD3 enrichment of *n* = 10 cells per condition is shown in **(F)**. Bars represent mean with SD. CD3 enrichment is defined as the ratio between CD3 intensity of parts of the cell on the spot vs. part of the cell covering the surrounding fibronectin. Statistical significance was tested with Kruskal–Wallis test with *post-hoc* Dunn's Multiple Comparison test. Scale bars represent 10 μm; ^*^p < 0.05; ^**^p < 0.01; ^***^p < 0.001.

Although CD6 has been recognized as a TCR co-receptor, the nature of the interaction with the TCR/CD3 complex has not been resolved. To provide a biophysical characterization of the interplay of these receptors during IS formation, we first studied CD6 and TCR/CD3 (co-)localization using antibody spots created with microcontact printing (Figure 1) (52). Wildtype Jurkat T lymphoma cells were seeded on microprinted antibody spots (5 μm in diameter) that were composed of 100% αCD6 or different concentrations of αCD3 (1-10-100%; diluted with mouse IgG2A-isotype control antibody) surrounded by fibronectin. Intracellular signaling through phospho-tyrosine (pTyr) was visualized by immunofluorescence staining on fixed cells (Figure 1A). Clustering of CD3 led to intracellular signaling as quantification of the pTyr fluorescence intensity levels in parts of the cells that covered the spots demonstrate a concentration-dependent increase on αCD3 spots (Figure 1B). Cells seeded on 1% αCD3 spots did not show any significant increase in pTyr levels compared to the 100% fibronectin (FN) spots (negative control) (Figure 1B). Next to clustering of CD3 also clustering of CD6 (using 100% αCD6 spots) induced T cell signaling; pTyr intensity on 100% αCD6 spots is comparable to the pTyr intensity on 10% αCD3 spots and significantly different from the intensity on 1% αCD3 and 100% FN spots. These results show that sufficient levels of cross-linking of CD3 or CD6 by microprinted antibody spots induce T cell signaling by increasing pTyr levels, an event generally leading to activation of Jurkat T cells.

To investigate the recruitment of CD6 upon cross-linking of the TCR/CD3 complex, we created Jurkat T cells stably expressing CD6-RFP or CD6-GFP. Total cell surface expression of CD6 as well as GFP and RFP expression in CD6-GFP cells, CD6-RFP cells and wildtype cells was determined with flow cytometry (Supplementary Figure 1). CD6-RFP Jurkat T cells were seeded onto the microprinted antibody spots (Figures 1C,D). As expected, confocal microscopy images show a strong recruitment of CD6-RFP to 100% αCD6 spots. Furthermore, a concentration-dependent recruitment of CD6-RFP was observed on 1–100% αCD3 antibody spots (Figure 1C). Quantification of CD6-RFP enrichment on the spots confirmed the concentration-dependent effect of αCD3 antibody in the spots on CD6-RFP enrichment. Also, CD6 enrichment to spots containing 100% αCD3 was comparable to that observed in the positive control, 100% αCD6 spots (Figure 1D). These results demonstrate that cross-linking of the TCR/CD3 complex induces recruitment of CD6 to spots. Consequently, we investigated whether cross-linking of CD6 also induces recruitment of the TCR/CD3 complex. To this end, wildtype Jurkat T cells were seeded on the microprinted antibody spots and stained for endogenous TCR/CD3 complex recruitment using a biotinylated αCD3 antibody and fluorescently labeled NeutrAvidin (Figures 1E,F). As expected, the TCR/CD3 complex was recruited to αCD3 spots, even at concentrations as low as 1% αCD3. Vice versa, CD3 was also recruited to 100% αCD6 spots, suggesting that the TCR/CD3 complex interacts with and co-migrates with CD6 (Figures 1E,F).

### CD3 ligation on micropatterned antibody spots causes immobilization of CD6

**Figure 2:**
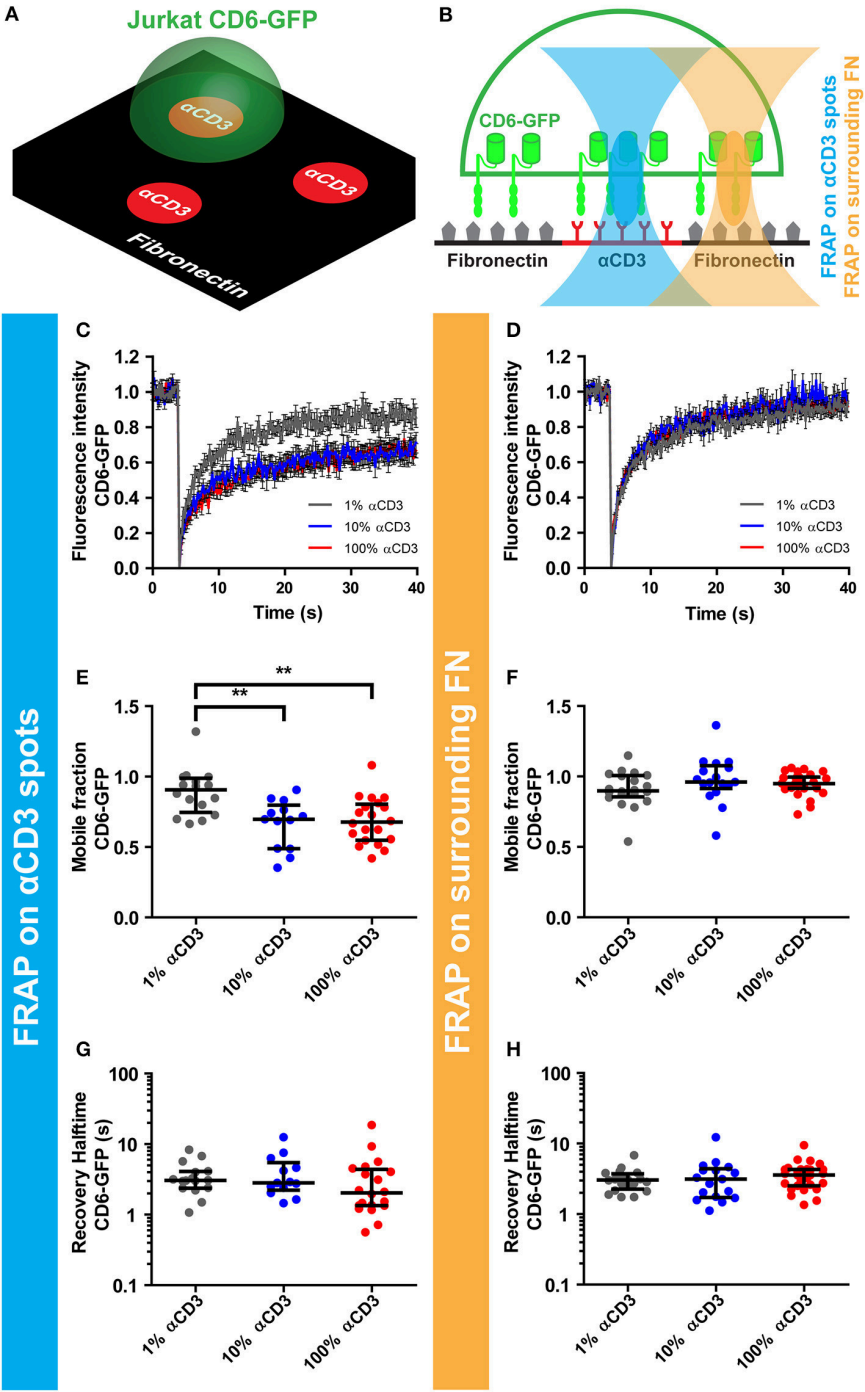
CD3 ligation on micropatterned antibody spots causes immobilization of CD6. **(A–H)** CD6-GFP Jurkat T cells were seeded on micropatterned substrates containing 100, 10, or 1% αCD3 spots, created using microcontact printing, surrounded by fibronectin. All spots were labeled with Alexa647. Schematic representation of the set-up of the experiment and positioning of the FRAP region are shown in **(A,B)** respectively. **(C–H)** FRAP was performed on CD6-GFP covering the antibody spots and on CD6-GFP covering surrounding fibronectin. Fluorescence intensity of CD6-GFP in the FRAP region on the antibody spots or on the surrounding fibronectin is shown in **(C,D)** respectively. Curves represent the mean of ≥13 measurements ± SEM. Individual FRAP curves were fitted with a single exponential model and values for the mobile fraction and the recovery half time for each separate curve were determined. Mobile fraction values on antibody spots and on surrounding fibronectin are shown in **(E,F)** respectively. Lines indicate median with the interquartile range represented as black bars. Statistical significance was tested with one-way ANOVA with *post-hoc* Tukey's Multiple Comparison test. Recovery halftime values on antibody spots and on surrounding fibronectin are shown in **(G,H)** respectively. Lines indicate median with the interquartile range represented as black bars. ^**^p < 0.01.

Next, we set out to investigate whether ligation of the TCR/CD3 complex on microprinted antibody spots influenced CD6 lateral mobility. To this end, CD6-GFP Jurkat T cells were seeded onto microprinted antibody spots composed of different concentrations of αCD3 (1-10-100%) (Figure 2A). To study CD6 mobility upon TCR/CD3 complex immobilization, fluorescence recovery after photobleaching (FRAP) of CD6-GFP was performed by bleaching circular 2.1 μm regions of interest (ROIs) both on spots and on fibronectin-coated areas surrounding these spots (Figure 2B). At the interface between cell and antibody spot-covered surface, FRAP revealed partial immobilization of CD6-GFP on 10 and 100% αCD3 spots, but not on 1% αCD3 spots (Figure 2C); a CD6-GFP fraction of ~30% was immobilized on 10 and 100% αCD3 spots, significantly different from the immobile CD6-GFP fraction on 1% αCD3 spots (appr. 10%), which was comparable to the immobile fraction on surrounding fibronectin-coated areas (Figures 2E,F). In comparison, FRAP outside antibody spot areas (on surrounding fibronectin-coated areas) showed unrestricted mobility of CD6-GFP with no effect of the αCD3 concentration within the spots (Figures 2D,F). The diffusion speed of the mobile CD6-GFP fraction was not affected by CD3 immobilization, as both on αCD3 spots and on surrounding fibronectin-coated areas recovery halftimes were similar (Figures 2G,H). These data indicate that a subpopulation of CD6 is immobilized upon CD3 ligation and confirm that CD6 at least partially interacts physically with the TCR/CD3 complex.

### CD6 is co-transported with TCR/CD3 in microclusters toward the cSMAC of the immunological synapse formed on αCD3-containing SLBs

When a T cells engages contact with a stimulating antigen-presenting cell, the TCR/CD3 complex is transported to the center of the immunological synapse (IS) formed at the interface between these cells. CD6 has been previously shown to co-localize with the TCR/CD3 complex in the central supramolecular activation cluster (cSMAC) of this IS (29, 35). However, techniques exploited so far have only shown static co-localization of CD6 and TCR/CD3 complex at a fully matured IS. Therefore, to investigate the dynamics of the CD6-TCR/CD3 complex interplay during IS formation, we studied synapse formation in Jurkat T cells seeded on planar supported lipid bilayers (SLBs), a well-established and widely used system to study early steps of IS formation (1, 3, 6, 11, 42). SLBs containing biotinylated lipids were prepared by spin coating lipids directly from chloroform solutions onto glass coverslips (45). Subsequently, ATTO647-labeled, biotinylated αCD3 antibody was coupled to the biotinylated lipids in the SLB via streptavidin, leading to free lateral diffusion of αCD3 antibody, confirmed by FRAP (data not shown).

**Figure 3:**
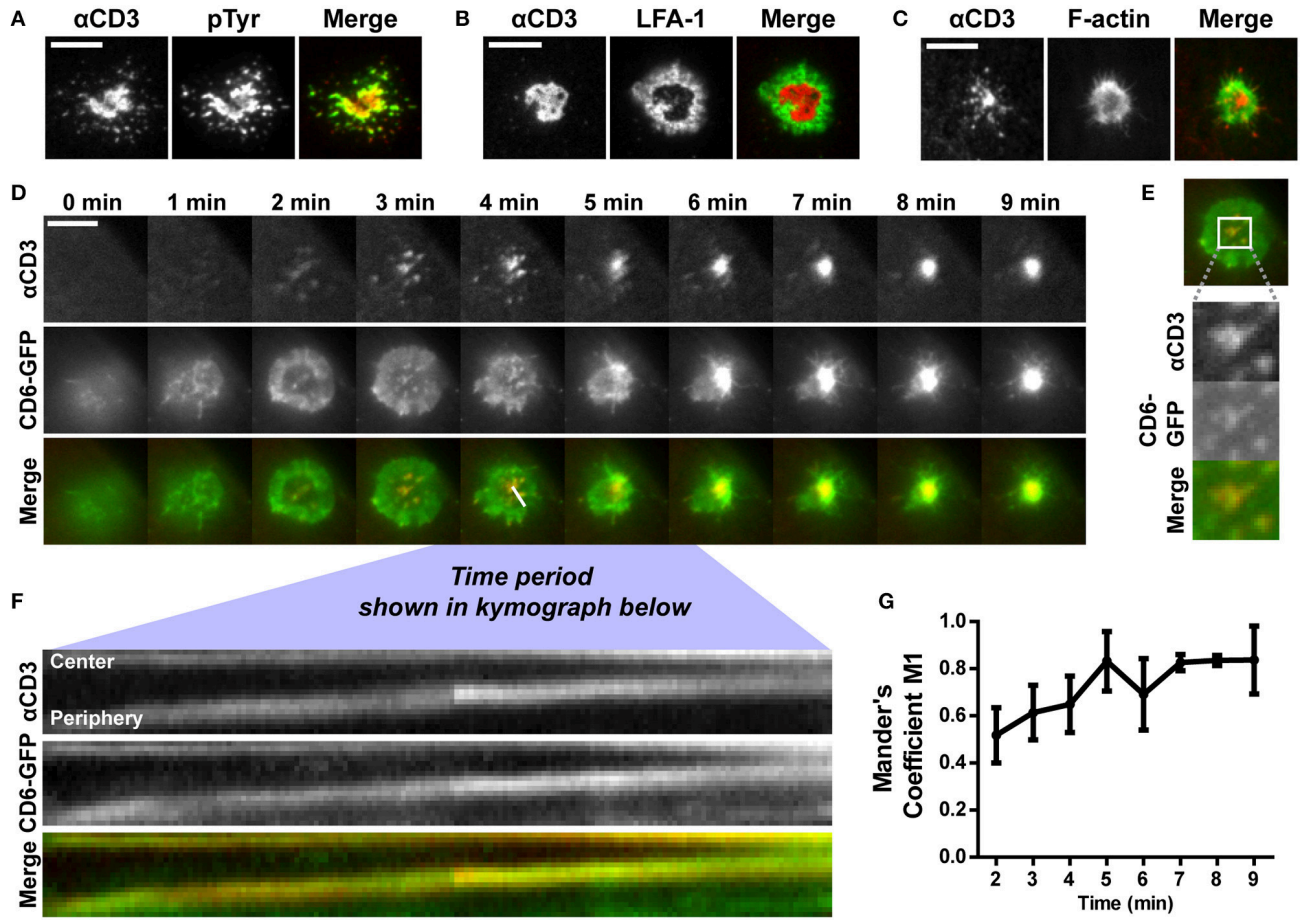
CD6 is co-transported with TCR/CD3 in microclusters toward the center of the immunological synapse formed on αCD3-containing SLBs. **(A,B)** Wildtype Jurkat T cells were seeded for 30 min on SLBs containing ATTO647-conjugated αCD3, and subsequently fixed and stained for immunological synapse markers. Representative TIRF microscopy images for phospho-tyrosine staining and LFA-1 staining (both labeled with Alexa488) are shown in **(A,B)** respectively. **(C)** LifeAct-GFP Jurkat T cells were seeded for 30 min on SLBs containing ATTO647-conjugated αCD3 and imaged using live cell imaging TIRF microscopy. Representative images are shown in **(C)**. **(D–G)** CD6-GFP Jurkat T cells were imaged while settling on a SLB containing ATTO647-conjugated αCD3, using TIRF microscopy live cell imaging. Snap shots of every minute of the time lapse are shown in **(D)**. Snap shots at time point 3 min, including a zoom-in of CD6-GFP and αCD3 microclusters are shown in **(E)**. Kymograph of the line (indicated in the Merge image at time point 4 min in **D**) during 3:45 to 6:12 min of the imaging period is shown in **(F)**. Co-localization over time starting at time point 2 min, represented as Mander's coefficient M1 (fraction of CD6-GFP overlapping with αCD3) in the central zoomed-in region indicated in **(E)** is shown in **(G)**. Scale bars represent 10 μm.

To assess whether Jurkat T cells formed an IS on these αCD3-containing SLBs, wildtype cells were allowed to interact with and spread on the SLBs. After fixation, the αCD3 antibody in the lipid bilayer was visualized to localize TCR/CD3 complexes. Representative brightfield images overlaying αCD3 signal are shown in Supplementary Figure 2. Wildtype Jurkat T cells were stained for phospho-tyrosine, to visualize signaling, and for integrin LFA-1, a classical component of the peripheral supramolecular activation cluster (pSMAC) surrounding the cSMAC in the IS (7). Furthermore, LifeAct-GFP Jurkat T cells were seeded onto SLBs to visualize the actin cytoskeleton (Figures 3A–C). Total Internal Reflection Fluorescence (TIRF) microscopy images of αCD3 in SLBs show that Jurkat T cells formed a large central cluster (cSMAC) containing TCR/CD3 in contact with SLBs (Figures 3A–C, left panels). Clustering of TCR/CD3 through αCD3 engagement in SLBs was able to mediate signaling as shown by the pTyr staining that co-localized with the αCD3 antibody in SLBs (Figure 3A). Staining of LFA-1 confirmed the formation of a typical peripheral ring (pSMAC) surrounding the cSMAC (Figure 3B). Also, typical exclusion of actin from the cSMAC region was seen in LifeAct-GFP Jurkat T cells on SLBs (Figure 3C). Overall, these data indicate that SLBs containing αCD3 allowed IS formation in interacting Jurkat T cells.

Next, we investigated the dynamics of CD6-TCR/CD3 interplay during synapse formation. To this end, Jurkat cells expressing CD6-GFP were imaged during spreading on and engagement of contact with SLBs using live cell TIRF microscopy (Figure 3D and Supplementary Video 1). Within 2 min after initial contact of the cell with the SLB, TCR/CD3 microclusters could be observed that were radially transported from the cell periphery toward the center of the cell-SLB interface (Figure 3D, top row). After 5 min a large, bright, and stable TCR/CD3-rich central cluster, the cSMAC of the IS, was formed on the SLB. During cell spreading in the first 3 min, CD6-GFP in the plasma membrane spread out and formed a peripheral ring-like pattern (Figure 3D, middle row). Within this ring, microclusters containing CD6-GFP were present, co-localizing to αCD3 microclusters formed in the SLB (Figure 3D, bottom row; Figure 3E). Kymograph analysis of the cross section indicated in the merged image at timepoint 4 min in Figure 3D (during 3 min and 45 s to 6 min and 12 s after initiation of cell-SLB contact) revealed that these microclusters, containing both CD6 and TCR/CD3, were transported from the periphery toward the central region (cSMAC) of the IS (Figure 3F). After 4 min the CD6-GFP ring started to disappear as a result of constant transport of microclusters toward the cSMAC. Thereafter a large, bright cluster of CD6-GFP was visible at the center of the cell-SLB interface, which largely co-localized with the TCR/CD3-rich cSMAC (Figure 3D, middle and bottom row). Intensity and co-localization analysis of the central part of the cell-SLB interface was performed for multiple cells (representative ROI is shown in Figure 3E). CD6-GFP signal intensity increased in the center of the cell over time, indicating continuous recruitment of CD6-GFP to the cSMAC (Supplementary Figure 3A). Also, co-localization of CD6-GFP and TCR/CD3 increased as the fraction of CD6-GFP overlapping with αCD3 (Mander's coefficient M1) increased over time (Figure 3G), as well as the Pearson coefficient and Mander's coefficient M2 (fraction of αCD3 overlapping CD6-GFP) (Supplementary Figures 3B,C). Of note, engagement of CD6 did not seem to affect IS formation, as pre-treatment and incubation of CD6-GFP Jurkat T cells with soluble human ALCAM-Fc did not lead to a difference in the fraction of cells forming a typical cSMAC within 30 min after seeding on SLBs (Supplementary Figure 4). Taken together, these data indicate that a fraction of CD6 molecules in the T cell membrane constantly associate with the TCR/CD3 complex from the very early moment of SLB engagement, until the formation of the mature cSMAC, where CD6-GFP is continuously being recruited. Thus, CD6 seems to be a member of the microclusters containing TCR/CD3, and is co-recruited with TCR/CD3 in these microclusters toward the IS.

### Disruption of actin polymerization inhibits TCR/CD3 and CD6 co-transport toward the cSMAC of the immunological synapse on αCD3-containing SLBs

**Figure 4:**
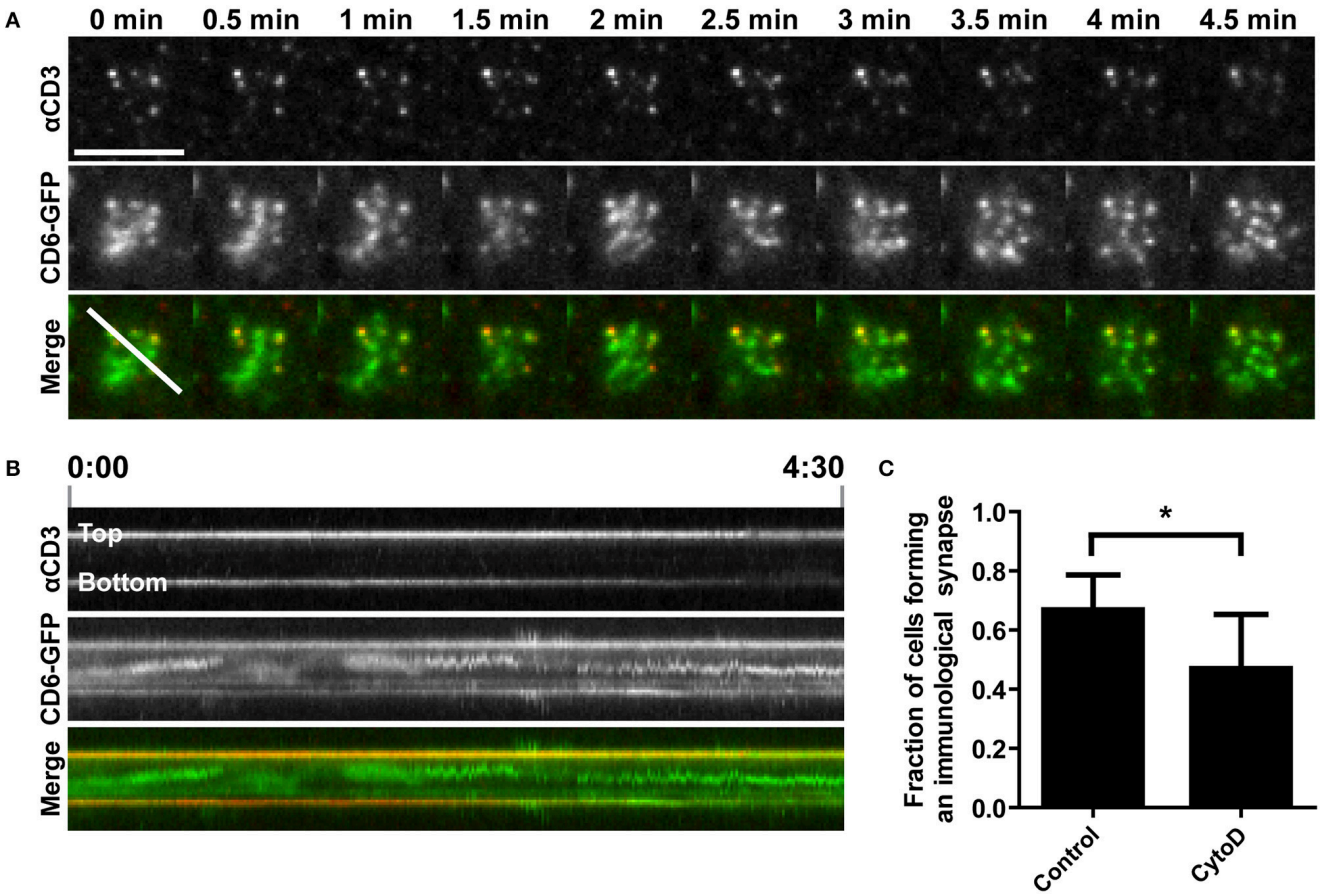
Disruption of actin polymerization inhibits TCR/CD3 and CD6 co-transport toward the cSMAC of the immunological synapse on αCD3-containing SLBs. **(A,B)** CD6-GFP Jurkat T cells, either untreated or pretreated with 0.5 μM Cytochalasin D for 15 min, were seeded on a SLB containing ATTO647-conjugated αCD3. Twenty minutes after seeding CD6-GFP and αCD3 dynamics were imaged using TIRF microscopy live cell imaging. Snap shots of every 30 s of the time lapse are shown in **(A)**. Kymograph of the line (indicated in the Merge image at time point 0 min in **A**) during the whole imaging period (4.5 min) is shown in **(B)**. **(C)** Wildtype Jurkat T cells, either untreated or pretreated with 0.5 μM Cytochalasin D for 15 min, were seeded for 30 min on SLBs containing ATTO647-conjugated αCD3, and subsequently fixed. Widefield microscopy was performed and cells (>44 cells per condition; three independent experiments) were scored for synapse formation based on identification of cells by brightfield displaying αCD3 positive cluster formation in a lipid bilayer (DiI) positive area. Average percentages of cells forming an immunological synapse are represented in **(C)**. Bars represent mean with SD. Statistical significance was tested with paired *t-*test. Scale bar represents 10 μm; ^*^p < 0.05.

The actin cytoskeleton provides a dynamic mechanical framework to spatially organize the IS, and the radial transport of TCR/CD3 microclusters is dependent on retrograde actin flow (10, 11, 53). To investigate whether transport of CD6 toward the cSMAC also depends on an intact actin cytoskeleton, Jurkat CD6-GFP cells were treated with 0.5 μM of the actin polymerization inhibitor cytochalasin D (CytoD) for 15 min before allowing them to interact with αCD3-containing SLBs. CD6 and TCR/CD3 microcluster formation and transport were imaged by TIRF microscopy (Figure 4 and Supplementary Video 2). In cytochalasin D-treated cells, microclusters of both CD6 and the TCR/CD3 complex were still formed after inhibition of actin polymerization (Figure 4A). However, these clusters were static and not transported toward the center of the cell-SLB interface, as in untreated cells shown in Figure 3D. Indeed, kymograph analysis shows that the position of peripheral clusters in the cross section indicated in the merged image at timepoint 0 min in Figure 4A is stable over time, as represented by the horizontal line in both the αCD3 and the CD6 channel (Figure 4B). In addition, some CD6 microclusters did not co-localize with TCR/CD3 microclusters. Although not completely immobile, these clusters did not move toward the center of the contact (Figure 4B). Moreover, treatment of cells with CytoD resulted in less Jurkat T cells forming a typical cSMAC within 30 min after SLB engagement and cell spreading (Figure 4C). This resulting difference may be an underestimation of the effect, as it is possible that CytoD-treated cells that did not engage the SLB at all have been washed away during fixation. In conclusion, these data demonstrate that the transport, but not the formation of CD6-TCR/CD3 microclusters clearly depends on actin polymerization.

### Interaction with αCD3-coated beads causes CD6 clustering and immobilization at cell-bead interface

**Figure 5:**
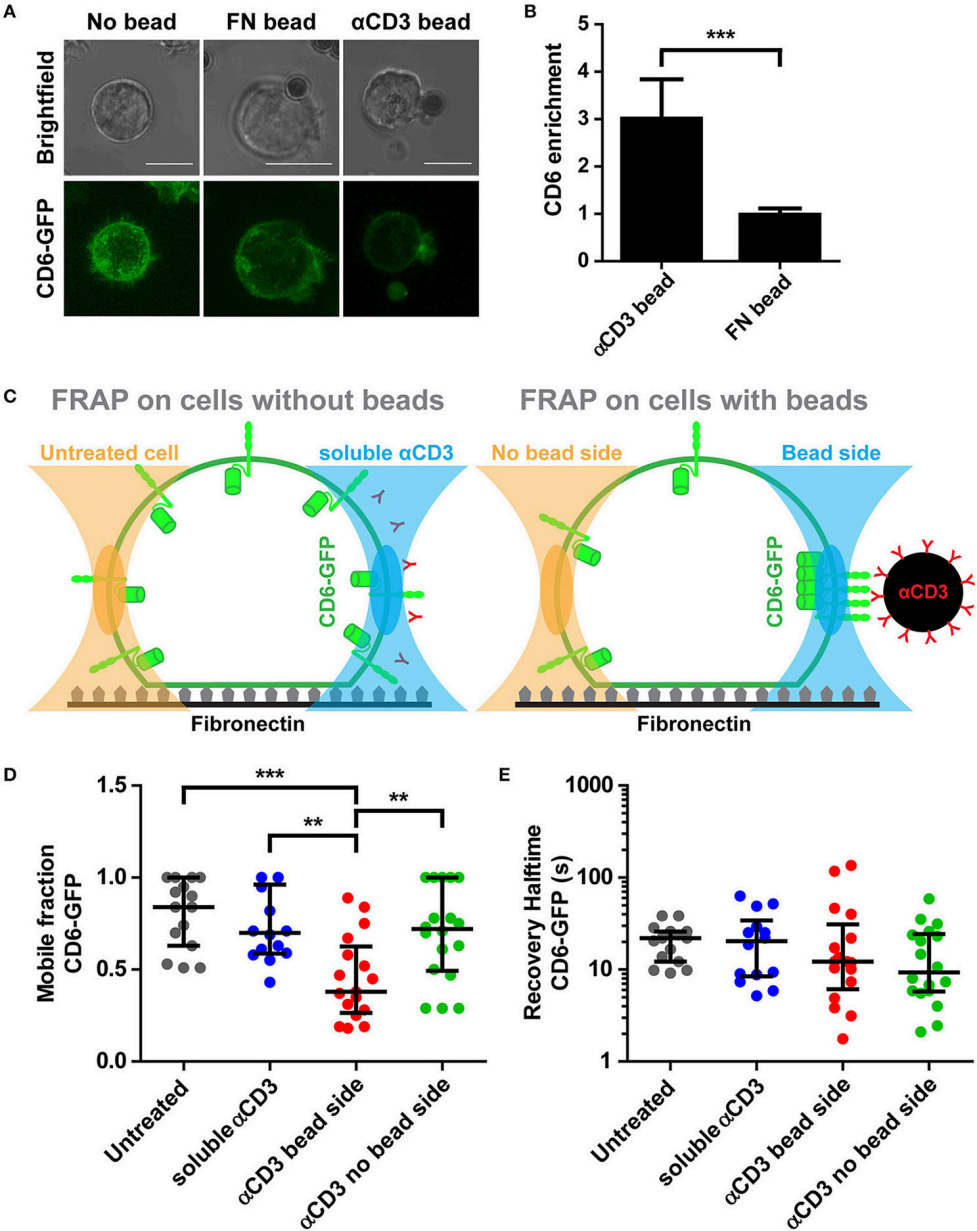
Interaction with αCD3-coated beads causes CD6 clustering and immobilization at cell-bead interface. **(A,B)** CD6-GFP Jurkat T cells together with fibronectin (FN)-coated or αCD3-coated beads were seeded on a fibronectin-coated surface. Representative brightfield and confocal fluorescence images of live cell imaging are shown in **(A)**. CD6-GFP images are brightest point reconstructions of z-stacks, allowing display of CD6 distribution of the entire cell. Quantification of CD6 enrichment at cell-bead interface is shown in **(B)**. CD6 enrichment (*n* = 10 cells per condition) is determined as the ratio between the area of cell that is in contact with the bead and an equal area at the opposite side of bead contact. Bars represent mean with SD. Statistical significance was tested with unpaired *t-*test. **(C–E)** CD6-GFP Jurkat T cells incubated with FN-coated or αCD3-coated beads or soluble αCD3 were seeded on a fibronectin-coated surface. Schematic representation of FRAP regions is depicted in **(C)**. FRAP was performed on CD6-GFP in cells with beads, at parts of the cell not in contact or in contact with the bead (respectively left and right in right panel in **C**) and compared with cells without beads, either untreated or treated with soluble αCD3 antibodies (respectively left and right in left panel in **C**). Individual FRAP curves (*n* ≥ 14 measurements per condition) were fitted with a single exponential model and values for the mobile fraction and the recovery half time for each separate curve were determined. Mobile fraction values and recovery halftime values of single FRAP curves for all conditions are shown in **(D,E)**, respectively. Lines indicate median with the interquartile range represented as black bars. Statistical significance was tested with one-way ANOVA with *post-hoc* Tukey's Multiple Comparison test. Scale bars represent 10 μm; ^**^p < 0.01; ^***^p < 0.001.

To better understand CD6 mobility in a cell-cell contact model, magnetic beads coated with αCD3 or with FN were added to CD6-GFP Jurkat T cells seeded on a FN-coated surface and CD6 enrichment at the cell-bead interface was determined. Brightest point reconstructions of confocal image stacks of CD6-GFP show that CD6 was a threefold more enriched to αCD3-coated beads than to fibronectin-coated beads (Figures 5A,B). Next, CD6 mobility was assessed using FRAP. FRAP measurements on CD6-GFP were performed on cells incubated with magnetic αCD3 beads, either at the cell-bead interface (bead side) or at the opposing free side of the cell (no bead side) (Figure 5C; FRAP on cells with beads). Of note, in this set-up, diffusion of CD6-GFP was assessed in a vertically oriented membrane and therefore 2 × 1 μm rectangular regions of interest (ROIs) were used for FRAP, in contrast to circular ROIs used on horizontal oriented membranes in Figure 2. As controls, CD6-GFP Jurkat T cells without beads, either untreated or incubated with soluble αCD3 were used for FRAP measurements (Figure 5C; FRAP on cells without beads). Resulting mobile fractions indicate that, as for CD6-GFP on 10 and 100% αCD3 antibody spots, a significant larger portion of the CD6-GFP population was immobilized at the cell-bead interface for cells in contact with αCD3-coated beads compared to CD6-GFP in the opposing side not in contact with a bead (Figure 5D). The mobile CD6-GFP fraction on the no bead site is comparable to that in untreated cells or cells treated with soluble αCD3. Again, the mobility of the mobile CD6-GFP fraction was not affected by interaction with the αCD3-coated bead, as recovery halftimes for all conditions did not differ significantly (Figure 5E). To determine the stability of this CD6-TCR/CD3 complex at the cell-bead interface, electromagnetic tweezers were used to displace the αCD3-coated bead through mechanical force (Supplementary Figure 5) (54). These data suggest that CD6 follows displacement of TCR/CD3 clusters and that the association between CD6 and TCR/CD3 complex is mechanically stable when exposed to mechanical forces of in the 200–900 pN range. Collectively these results confirm previous observations on microprinted antibody spots: cross-linking the TCR/CD3 complex by immobilized αCD3 results in immobilization of a significant fraction of CD6-GFP molecules, which strongly indicates a stable interaction between CD6 and the TCR/CD3 complex.

## Discussion

In this study, we applied different experimental techniques to characterize the interplay between CD6 and the TCR/CD3 complex. We show that CD6 and the TCR/CD3 complex are co-recruited to stable stimulatory clusters, both in Jurkat T cells seeded on antibody spots and in Jurkat T cells in contact with αCD3-coated beads. This association to TCR/CD3 applies to only a fraction of the CD6 population, as FRAP measurements on CD6-GFP (both in cells on αCD3 antibody spots or in cells in contact with αCD3-coated beads) indicate that more than half of the CD6-GFP population was still mobile. If the interaction was transient, a reduction in recovery time but no change in immobile fraction would have been expected. This partial association of CD6 with TCR/CD3 confirms previous reports by Gimferrer and colleagues which showed a partial association using co-precipitation (35). Although substantial, the fully mobile and non-associated fraction CD6 of ~70% reported here may be an overestimation, as we made use of an over-expression model that most probably leads to an excess of CD6. This interaction between CD6 and TCR/CD3 seems mechanically rather stable, as we could show that CD6 follows displacement of TCR/CD3 by moving αCD3-coated magnetic beads with electromagnetic forces of 200–900 pN.

Next to recruitment to static ligands, we exploited SLBs where αCD3 could freely diffuse in the lateral plane. This setup allowed us to visualize CD6 dynamics during IS formation. We found that CD6 co-localizes with TCR microclusters on the Jurkat T cell membrane during IS formation. These CD6-TCR/CD3 microclusters were transported toward the cSMAC of the IS, which finally resulted in CD6-TCR/CD3 co-localization in the mature IS, as reported previously by us and others (29, 35). Since it has been shown that TCR signaling predominantly takes place in these microclusters that localize outside the cSMAC (1, 2, 12), the presence of CD6 in these microclusters suggests a role for CD6 in TCR receptor (co-)signaling. In our SLBs no ligand for CD6 was present; the co-localization of CD6 with TCR/CD3 microclusters we have demonstrated in this study is therefore independent of direct CD6 ligand binding. Therefore, although CD6-ALCAM interactions have been shown to localize to the cSMAC in stable T cell-DC interactions (29, 35), we cannot exclude that ligand binding affects the preceding CD6-TCR/CD3 co-localization in microclusters during IS formation and transport toward the cSMAC. Whether TCR/CD3 and CD6 interact directly or indirectly remains to be determined. Direct interaction between CD6 and TCR/CD3 is deemed unlikely, as the dimensions of receptor-ligand interactions differ; the optimal distance for TCR-pMHC is calculated to be 14–15 nm, whereas the binding distance between CD6 and ligand ALCAM would be probably larger than 21 nm (31).

Furthermore, in all set-ups we have used antibodies directed against CD3 to induce TCR/CD3 clustering and triggering. Although this is an artificial way of inducing T cell activation, it has been shown that stimulating CD3, without presence of an MHC-antigen complex, can sufficiently induce IS formation in Jurkat T cells (11). Furthermore, it has been shown that CD6 is phosphorylated on its cytoplasmic tail upon cross-linking of CD3 and CD2/CD3 co-cross-linking (34). TCR/CD3 complex triggering using αCD3 antibodies may result in differential downstream signaling than triggering with specific peptide-MHC complexes. As the association between CD6 and TCR/CD3 may depend on phosphorylation of CD3 and/or CD6 and could lead to different proteins interacting with CD3 and/or CD6, the type of molecule triggering the TCR/CD3 complex (αCD3 or pMHC complex) might modulate the CD6-TCR/CD3 interaction. Investigation of co-localization of CD6 cytoplasmic tail mutants with TCR/CD3 microclusters during IS formation would be able to shed more light on this question.

The actin cytoskeleton provides a dynamic mechanical framework to spatially organize the IS, and the radial transport of TCR microclusters depends on retrograde actin flow (10, 11, 53). Also in our set-up actin was present in a peripheral ring on the intracellular side of the IS and excluded from the cSMAC. Furthermore, we found that the CD6-TCR/CD3 co-transport in microclusters toward the cell center depends on actin polymerization. This suggests that CD6, similar to the TCR/CD3 complex, is linked to the actin cytoskeleton. Moreover, the peripheral ring-like pattern of CD6 we saw during the initial cell spreading is reminiscent of the F-actin pattern observed in other studies on T cells forming an IS (11). The actin cytoskeleton itself might even provide the link between CD6 and TCR/CD3. Interestingly, CD6 has been shown to associate with the adaptor protein SLP-76, which is part of TCR microclusters (13, 33, 55). TCR-induced tyrosine phosphorylation of SLP-76 has been shown to be important in the recruitment of the proteins Nck and WASp to TCR microclusters for actin polymerization (56). However, CD6 association to the TCR/CD3 complex through SLP-76 cannot explain CD6-TCR/CD3 co-transport into the cSMAC, as it has been shown that SLP-76 (together with ZAP70) dissociates from TCR microclusters before these coalesce with the cSMAC, and localizes to unidentified perinuclear structures (13, 55).

Next to SLP-76, CD6 also interacts with the actin-binding adaptor protein syntenin-1 (57). Syntenin-1 has been implicated in functional asymmetry in T cells and actin polymerization and accumulation in T cell activation (58, 59). Presence of syntenin-1 is needed for CD3 accumulation at the cSMAC (59), and may provide the link between CD6 and the TCR/CD3 complex, in this way facilitating CD6-TCR/CD3 microcluster transport toward the cSMAC. Unlike SLP-76, syntenin-1 has been shown to localize to the cSMAC of the IS, where it co-localizes with CD6 and TCR/CD3 (57). Any possible link between CD6 and the TCR/CD3 via syntenin-1 would, however, be independent of the actin cytoskeleton, as the cSMAC is devoid of actin (11).

Detailed investigation of the organization of CD6, TCR/CD3, SLP-76 during IS formation using super-resolution imaging, such as Sherman and colleagues showed for the TCR, LAT, ZAP-70, and SLP-76 (60), could provide more insight into the organization of these TCR-CD6 microclusters and the exact role of SLP-76 and syntenin-1 in the interaction between these cell surface receptors. Next to that, super-resolution microscopy would also be able to shed light on the hitherto open question whether CD6 and TCR/CD3 associate directly or indirectly at the steady state level in the cell membrane of a resting T cell. New possible interaction partners of CD6 are still being identified and crucial molecules linking CD6 to the TCR/CD3 complex at the steady state level and/or during IS formation may therefore be still unknown at present (61).

Although the data presented here further substantiate the interplay between CD6 and TCR/CD3 and indicate that this co-recruitment already occurs in TCR microclusters prior to stable IS formation, it still remains a subject of debate whether CD6 signaling plays a stimulatory or inhibitory role in T cell activation. On the one hand, many studies employing monoclonal antibodies or soluble CD6 to target CD6 or its interaction with ALCAM have underlined the stimulatory role of CD6 in T cell activation and proliferation (23–29). On the other hand, the mere presence of CD6 in the T cell membrane has inhibitory effects on calcium response and IL-2 release in TCR-activated Jurkat T cells (30). Also, CD6 associates with family member CD5 (19), an established inhibitor of T cell signaling (62), which may in fact indirectly give CD6 its inhibitory capacities (31). It has been proposed that CD6 acts as a decoy receptor to capture downstream signaling molecules away from the TCR signaling complex, as it localizes to the cSMAC of the IS, an area where TCR signaling is terminated through TCR endocytosis and degradation (31, 63). However, our data show that preceding formation of a stable mature IS, CD6 already co-localizes with TCR/CD3 microclusters, which are believed to be stimulating T cell activation. Still, CD6 may compose its own signaling hub independent of the TCR/CD3-LAT signalosome, as a proteomics study by Roncagalli and colleagues showed that LAT is dispensable for CD6-SLP-76 association (64). Because the CD6 gene has been shown to subject to alternative splicing upon T cell activation, the role of CD6 may alter during T cell-APC interaction, as one of the alternatively spliced forms has been shown to no longer translocate to the IS (65–67). In this study we have made use of full-length CD6 in our over-expression models. Therefore, investigation of the localization of alternatively spliced CD6 forms during IS formation, together with functional read-outs such as calcium fluxing and T cell proliferation, might provide more insight in the role of CD6 in microcluster formation and the mature IS.

## Data availability statement

The raw data supporting the conclusions of this manuscript will be made available by the authors, without undue reservation, to any qualified researcher.

## Author contributions

MM, AC, and CF designed the study. MM performed microcontact printing experiments. BJ, JW, and RB assisted with microcontact printing. SM performed lipid bilayer experiments. BJ assisted in cell culture, lipid bilayer experiments and image analysis. FC and JK performed magnetic tweezer and beads experiments. MM, SM, and FC analyzed data with support of JtR. SM, MM, AC, and CF wrote manuscript, with input from all authors.

### Conflict of interest statement

The authors declare that the research was conducted in the absence of any commercial or financial relationships that could be construed as a potential conflict of interest.
