# Supplementary material for: Biophysical Characterization of CD6—TCR/CD3 Interplay in T Cells
Source: Front Immunol. 2018 Oct 9;9:2333. doi: 10.3389/fimmu.2018.02333 (PMC6189472; doi:10.3389/fimmu.2018.02333)
Supplement: Supplementary file 3 [file Data_Sheet_1.pdf]

Supplementary Material

Biophysical Characterization of CD6 – TCR/CD3 interplay in T cells

Marjolein B.M. Meddens[#], Svenja F.B. Mennens[#], F. Burcu Celikkol, Joost te Riet, Johannes S. Kanger, Ben Joosten, J. Joris Witsenburg, Roland Brock, Carl G. Figdor[*] and Alessandra Cambi[*]

[#]*authors contributed equally to this work*

*** Correspondence:**
Alessandra Cambi
Alessandra.Cambi@radboudumc.nl

Carl Figdor
Carl.Figdor@radboudumc.nl

1 Supplementary Methods

1.1 Flow cytometry

The cell surface expression of CD6 in wildtype Jurkat T cells, CD6-GFP Jurkat T cells and CD6-RFP Jurkat T cells was assessed using flow cytometry. In short, cells were taken from culture and washed in PBA (PBS/0.5%BSA/0.01%NaN3) and subsequently blocked in PBA with 2% human serum for 30 min at 4°C. Subsequently cells were stained with Mouse IgG1 anti-human CD6 (M-T605; BD Biosciences) in PBA for 30 min, washed with PBS and finally stained with secondary antibody Goat-anti-Mouse(H+L)-Alexa647 (Invitrogen) in PBA for 30 min. GFP and RFP expression was determined in unstained cells. Cells were fixed with 4% PFA in PBS for 10 min at RT and resuspended in sheath fluid before measurement. Samples were measured on a CyAn ADP (Beckman Coulter). Analysis was performed with Flowjo Software (Treestar Inc) version X.0.7. The cell population was separated from debris by gating in the Forward/Sideward Scatter Plot. All gated cells (>10,000 cells in each sample) were subsequently analyzed for CD6, RFP and GFP expression.

1.2 Immunofluorescence

For images showing cell outlines in brightfield and the αCD3 signal in cells seeded on supported lipid bilayers (SLBs), Jurkat T cells resuspended in Hank's Balanced Salt Solution (HBSS; Gibco) were seeded on SLBs for 30 min at 37°C at a cell concentration of 400,000 cells per ml. Samples were washed with PBS and subsequently fixed with 4% PFA in PBS for 15 min at RT.

After fixation, samples were washed once with PBS, after which they were imaged at a Leica DMI6000 widefield microscope equipped with a HC PL APO 63x 1.40 NA oil immersion objective.

For the experiment studying the effect of binding of CD6 by soluble ALCAM-Fc on IS formation, recombinant human ALCAM-Fc chimera (656-AL) from R&D Systems was used. CD6-GFP Jurkat cells were taken from culture and incubated in HBSS with or without 10 µg/ml soluble ALCAM-Fc for 30 min at 4°C at a concentration of 800,000 cells per ml. Next, cell suspensions were added onto αCD3-containing SLBs, reaching a final cell concentration of 400,000 cells per ml. Samples were incubated for 30 min at 37°C. After incubation, samples were fixed with 4% PFA in PBS for 15 min at RT. Finally, samples were washed once and imaged in PBS directly after preparation. CD6-GFP, αCD3-ATTO647, DiI and brightfield signals were imaged with TIRF microscopy at an Olympus IX-71 wide field fluorescence microscope equipped with a 3-line TIRF system and a Hamamatsu ImagEM EM-CCD camera equipped with a PL APO 60×/1.4 NA oil immersion TIRF objective. The fraction of cells forming an immunological synapse on SLBs upon treatment was determined by manual counting. Cells having formed an immunological synapse were defined as CD6-GFP positive cells, also visible in brightfield, on top of supported lipid bilayer (DiI-positive area), overlaying an αCD3 positive cluster.

1.3 Bead displacement with magnetic tweezers

The electromagnetic tweezers are composed of an iron rod (radius 100 µm) with a 250 Ampere/turns electric coil around it (1). The magnetic tip is positioned using a 3D micromanipulator (Narishige, Japan). Magnetic tweezers were force calibrated using drag force of beads in viscous media. The force exerted on a 4.5 µm particle with 1 Volt supplied tweezers was determined as 2.7 nN when the tweezer-bead distance was 20 µm, decreasing to 200 pN when the tweezer-bead distance was 100 µm. In the experiments, supplied voltage was adjusted between 1-2 Volts as bead-tweezer tip distance varied between 100-15 µm, resulting in pulling forces from 200 pN to 900 pN. Dynal CD3 beads coated with mouse monoclonal anti-CD3 antibody (Invitrogen) with a diameter of 4.5 µm were used for bead displacement experiments. Jurkat CD6-GFP cells were seeded on fibronectin-coated coverslips in imaging medium (RPMI 1640, 25 mM HEPES, 0.5% BSA) and subsequently beads were added to the cells in a concentration of 5 µM. Once the beads were in contact with the cell membrane, beads were pulled along the cell membrane with the electromagnetic tweezers. Imaging of cells with beads was performed on a Zeiss LSM510 meta confocal laser scanning microscope equipped with a PL APO 63×/1.4 NA oil immersion objective. Cells were imaged at RT to slow down internalization of the beads. Time-lapse recordings of cells in the brightfield and confocal fluorescence (CD6-GFP) channels were taken at a time interval of 6 seconds between frames during a total of 15 min of bead displacement.

2 Supplementary Figures

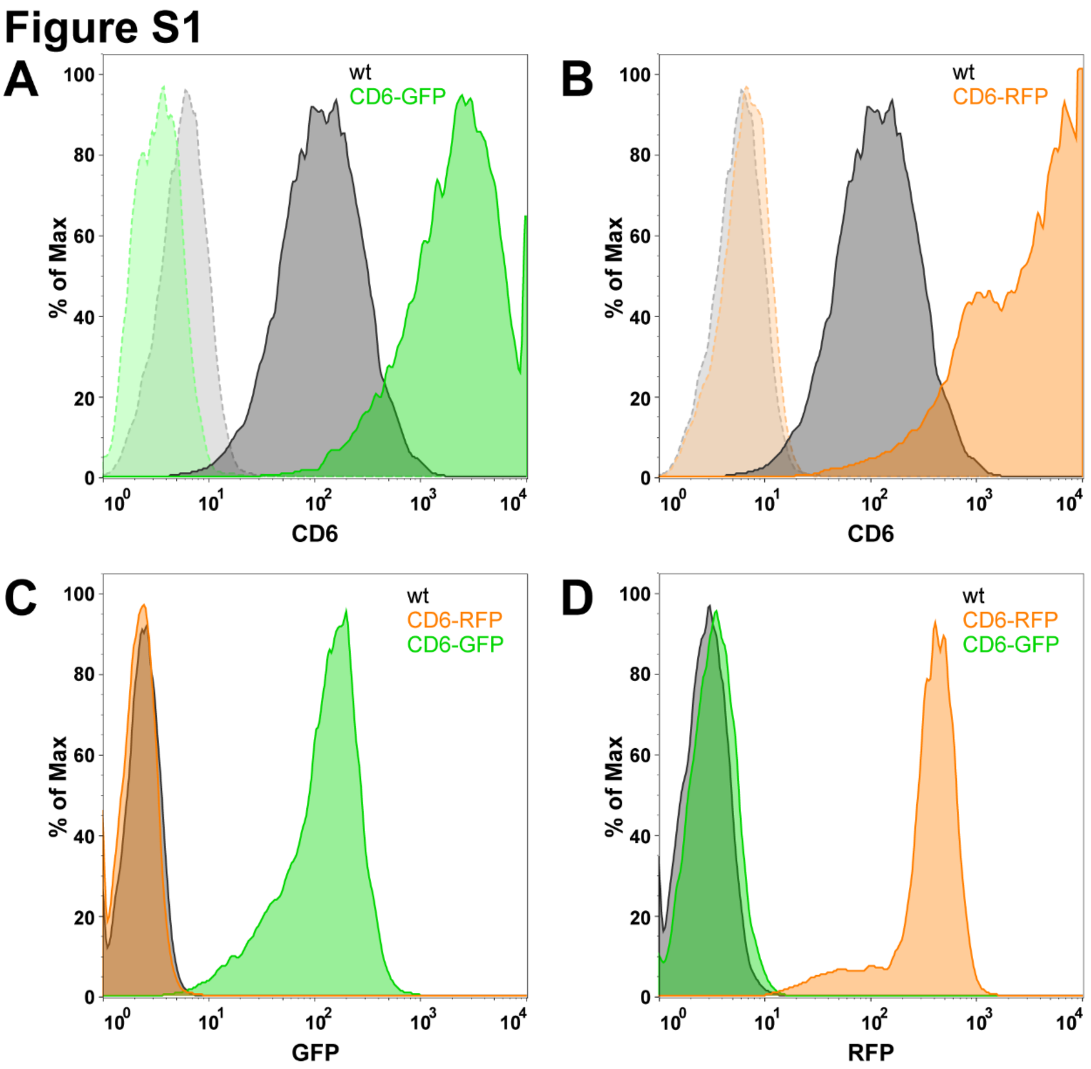

Supplementary Figure 1. Expression of CD6, CD6-GFP and CD6-RFP in Jurkat T cell lines.

(**A**-**B**) Representative fluorescence intensity profiles of CD6 expression determined with anti-CD6 antibody staining and measured with flow cytometry in CD6-GFP vs. wildtype Jurkat T cells (**A**) and CD6-RFP vs. wildtype Jurkat T cells (**B**). Grey indicates wildtype Jurkat T cells (wt), green indicates CD6-GFP Jurkat T cells, orange indicates CD6-RFP Jurkat T cells. Dashed graphs indicate appropriate isotype controls. (**C**-**D**) Representative fluorescence intensity profiles of GFP expression (**C**) and RFP expression (**D**) measured with flow cytometry in CD6-GFP Jurkat T cells, CD6-RFP Jurkat T cells and wildtype Jurkat T cells. Grey indicates wildtype Jurkat T cells (wt), green indicates CD6-GFP Jurkat T cells, orange indicates CD6-RFP Jurkat T cells.

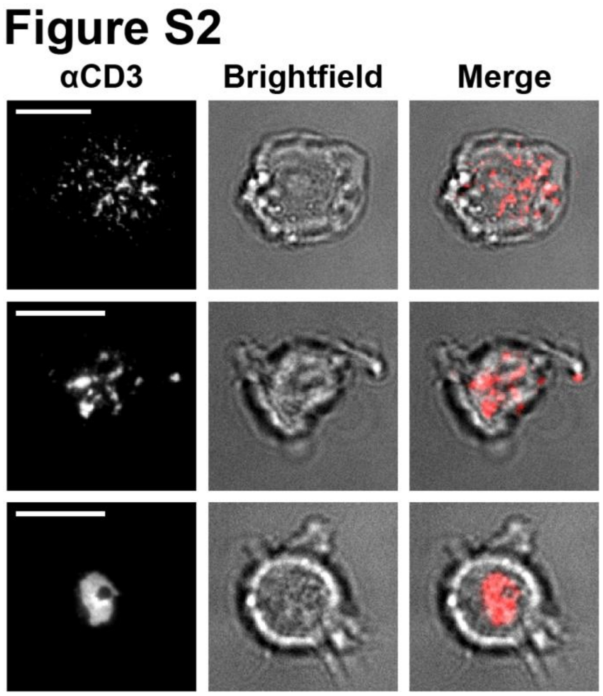

Supplementary Figure 2. αCD3-containing SLBs induce immunological synapse formation at the T cell-SLB interface.

Representative images of αCD3 and brightfield showing Jurkat T cells at different stages of immunological synapse formation after seeding of 30 min on SLBs containing ATTO647-conjugated αCD3. Scalebars represent10 µm.

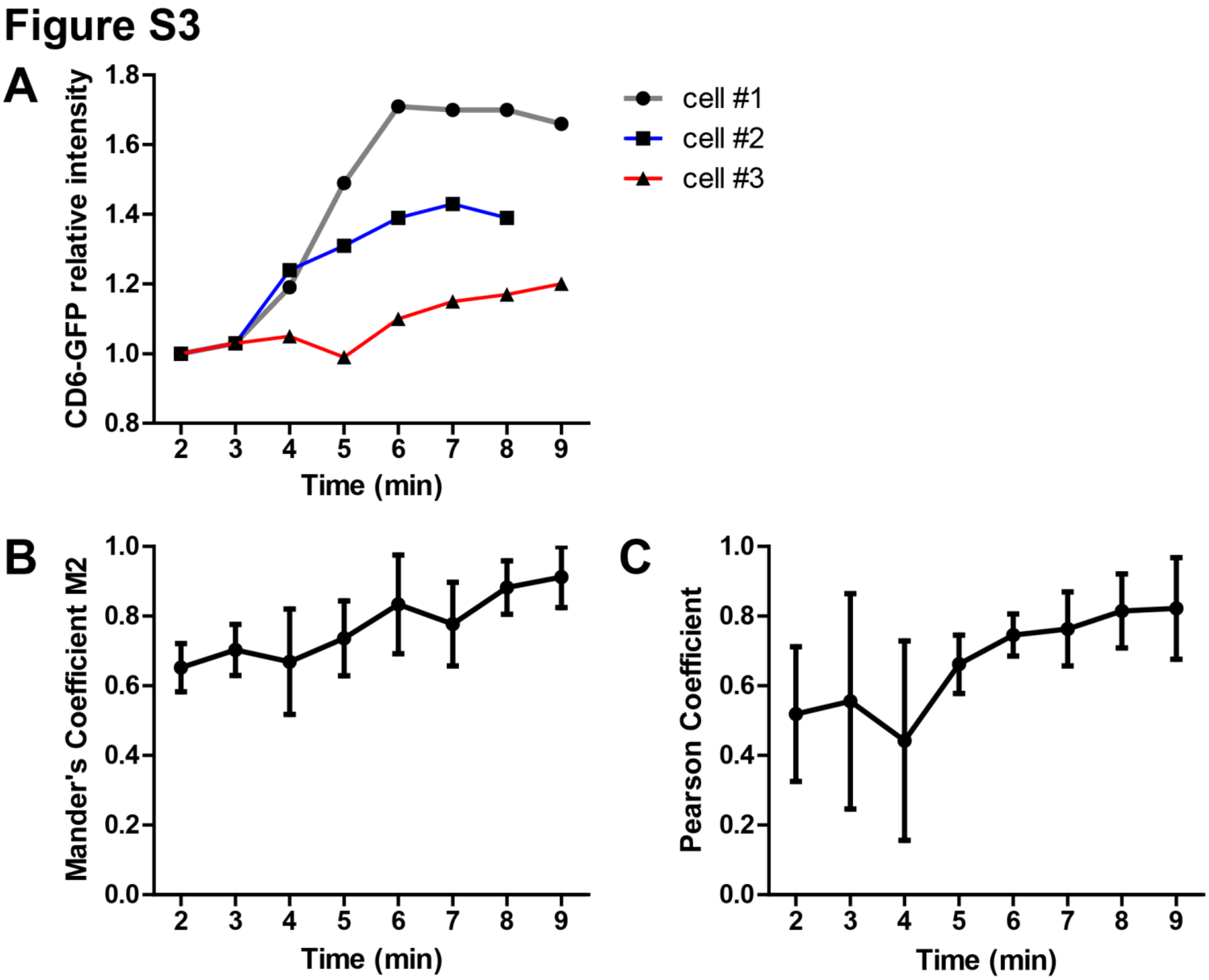

Supplementary Figure 3. CD6-GFP signal intensity and CD6-GFP and TCR/CD3 co-localization increase during immunological synapse formation.

TIRF microscopy live cell imaging data of CD6-GFP Jurkat T cells forming an immunological synapse on a SLBs containing ATTO647-conjugated αCD3 were analyzed for CD6-GFP signal intensity and co-localization (n = 3 cells). Representative images of one cell (cell #1) are shown in Figure 3D. A region of interest (ROI) of the central part of the cell-SLB interface (indicated in Figure 3F) was analyzed from the moment of TCR/CD3 microcluster appearance (t = 2 min in Figure 3D). (**A**) CD6-GFP signal intensity (integrated density) in the central ROI over time, relative to the ROI signal intensity (integrated density) at t = 2 min. Signal intensity is indicated separately for the 3 cells analyzed. (**B**-**C**) Average Mander's coefficient M2 (fraction TCR/CD3 overlapping CD6-GFP) (**B**) and average Pearson Coefficient (**C**) over time (n = 3 cells) in the central ROI. Graphs represent mean with SD.

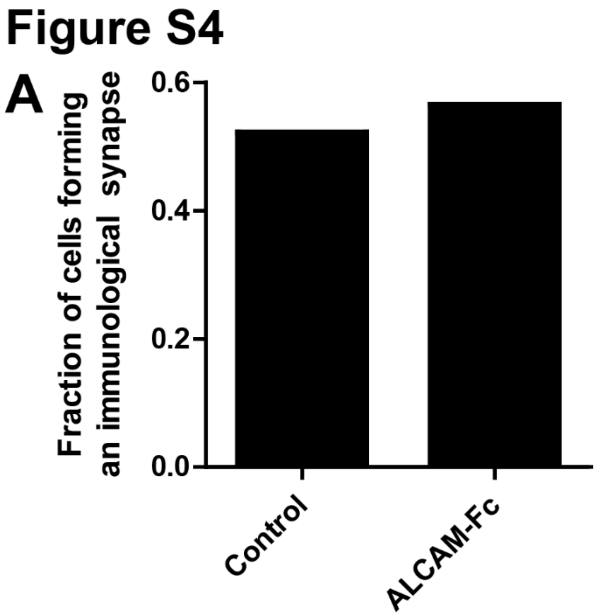

Supplementary Figure 4. CD6 engagement by soluble ALCAM does not prevent immunological synapse formation on αCD3-containing SLBs.

CD6-GFP Jurkat T cells, either untreated or pretreated with 10 µg/ml soluble ALCAM-Fc for 30 min, were seeded for 30 min on SLBs containing ATTO647-conjugated αCD3, and subsequently fixed. Widefield microscopy was performed and cells (>23 cells per condition) were scored for synapse formation based on identification of CD6-GFP positive cells, also visible in brightfield, displaying αCD3 positive cluster formation in a lipid bilayer (DiI) positive area. Bars represent fraction of cells forming an immunological synapse in ALCAM-Fc treated cells (ALCAM-Fc) or untreated cells (Control).

Figure S5

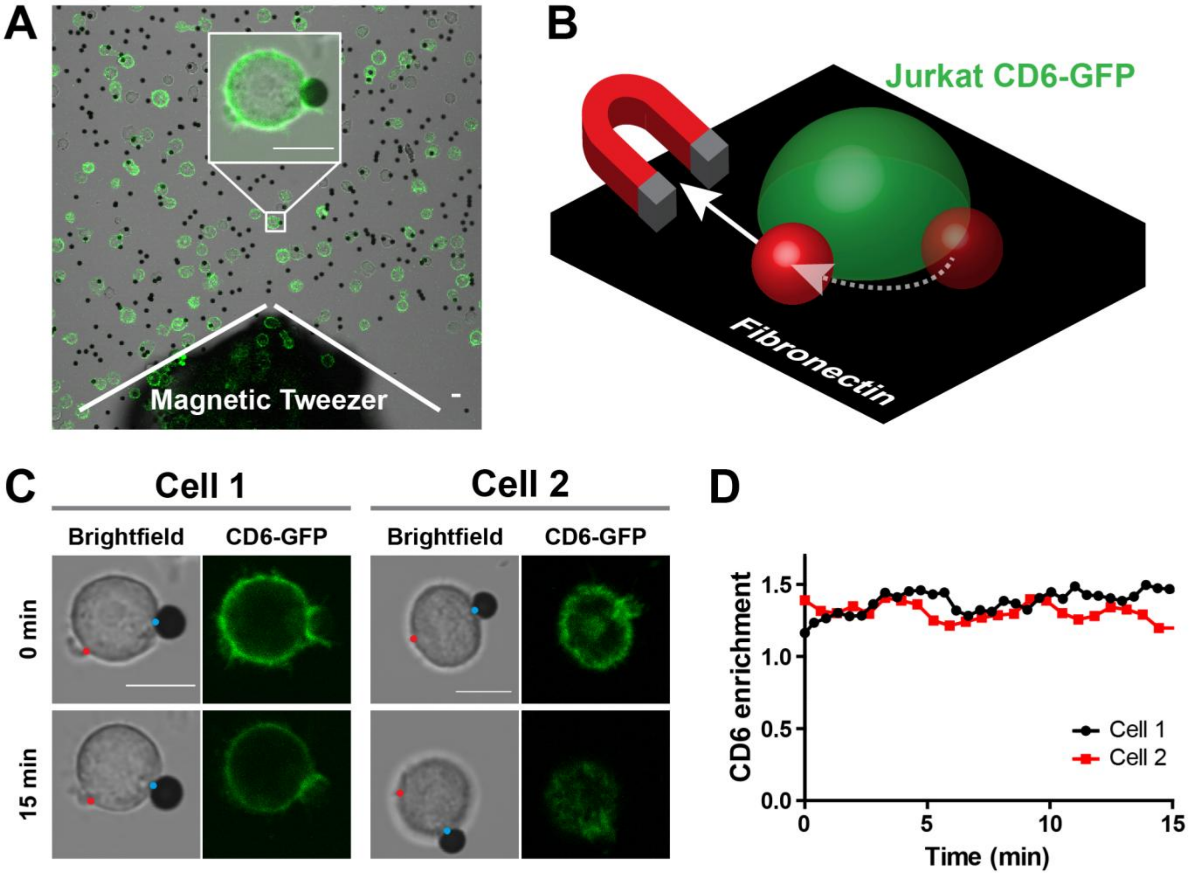

Supplementary Figure 5. CD6 enrichment at cell-bead interface follows αCD3-coated bead displacement upon application of electromagnetic force.

(**A**-**D**) CD6-GFP Jurkat T cells were seeded on a fibronectin-coated surface and incubated with αCD3-coated magnetic beads. A representative overview image, including zoom-in, of cells in with beads, indicating CD6-GFP in green, is shown in (**A**). Once the beads were in contact with the cell membrane, beads were pulled along the cell membrane with electromagnetic tweezers. Images were taken with 6 second intervals during the total 15 min of bead displacement using live cell brightfield and confocal fluorescence microscopy. Schematic representation of the bead pulling set-up is shown in (**B**). Representative brightfield and single confocal images with bead in focus before (t = 0 min) and after bead pulling (t = 15 min) are shown in (**C**). Cell rotation during pulling was accounted for by tracking position of cell features, indicated with red and blue dots. Travelled distance for the beads is 3 µm and 8 µm for Cell 1 and 2, respectively. CD6 enrichment at the cell-bead interface determined during the bead displacement, defined as the ratio of CD6-GFP intensity in the region of cell membrane around the bead and an equal sized area at the opposite side of the cell, is shown in (**D**). Scale bars represent 10 µm.

3 Supplementary References

1. Shekhar S, Klaver A, Figdor CG, Subramaniam V, Kanger JS. Spatially resolved local intracelullar chemical sensing using magnetic particles. Sensors and Actuators B: Chemical. 2010;148(2):531-8.
